# The Effect of Menstrual Cycle on Perceptual Responses in Athletes: A Systematic Review With Meta-Analysis

**DOI:** 10.3389/fpsyg.2022.926854

**Published:** 2022-07-13

**Authors:** Ana Carolina Paludo, Armin Paravlic, Kristýna Dvořáková, Marta Gimunová

**Affiliations:** ^1^Incubator of Kinanthropology Research, Faculty of Sports Studies, Masaryk University, Brno, Czechia; ^2^Faculty of Sport, University of Ljubljana, Ljubljana, Slovenia; ^3^Science and Research Centre Koper, Institute for Kinesiology Research, Koper, Slovenia; ^4^Department of Health Promotion, Faculty of Sports Studies, Masaryk University, Brno, Czechia; ^5^Department of Kinesiology, Faculty of Sports Studies, Masaryk University, Brno, Czechia

**Keywords:** athletes, behavior, female, menstrual cycle, ovarian hormones

## Abstract

This article aimed to investigate the effects of menstrual cycle phases on perceptual responses in athletes by means of systematic review and meta-analysis. The search was conducted in the PubMed, Web of Science, and Sport Discus databases considering articles with two or more menstrual phases for comparison. The PECO criteria were used for the keywords “menstrual cycle,” “athletes,” and “perceptual responses” with their respective entry terms. Of 1.165 records identified, 14 articles were available for the final evaluation, while eight articles were eligible for a meta-analysis. The perceptual responses evaluated in the studies were: motivation, competitiveness, sleep quality, stress, muscle soreness, fatigue, perceived effort, mood, menstrual symptoms, perceived endurance, and readiness. The meta-analysis was conducted for perceived effort only. The results showed that the level of perceived exertion does not differ two phases of the menstrual cycle (MD = 3.03, *Q* = 1.58, *df* = 1, *p* = 0.209), whereas RPE was 19.81 ± 0.05 and 16.27 ± 0.53 at day 1–5 and day 19–24, respectively. Two studies found statistically significant changes in motivation and competitiveness during the cycle, with better outcomes in ovulatory phase compared to follicular and luteal. One study found an increase in mood disturbance in the pre-menstrual phase (vs. mid-cycle); one decreased vigor in the menstrual phase (vs. luteal); one increased the menstrual symptoms in the follicular phase (vs. ovulation), and one study reported increased fatigue and decreased sleep quality on luteal phase (vs. follicular). The remaining studies and variables were not affected by the menstrual cycle phase. Based on the results from the studies selected, some perceptual responses are affected in different menstrual cycle phases. A “favorable” subjective response in athletes was noticed when the ovarian hormones present an increase in concentration levels compared to phases with lower concentration. Different perceptual variables and methodological approaches limit the generalization of the conclusion.

## Introduction

Lately, there has been a great interest in the influence of the menstrual cycle on women's athletic performance. Thus, sports professionals are investigating different strategies that could be implemented into their practice to monitor the female athlete's performance while considering individual athletes' responses to training loads through different phases of the menstrual cycle (De Jonge et al., 2019; Carmichael et al., 2021a; Meignié et al., 2021). Professional female teams and organizations started to monitor responses such as sleep, recovery and performance perception in order to develop individual training strategies to address possible changes during the menstrual cycle and improve the athletes' ability to train (Carmichael et al., 2021b).

The menstrual cycle, on average, lasts 28 days (range 21–35 days) (Lenton et al., 1984) and can be separated into two distinct phases, namely the follicular and luteal, which are separated by the ovulation period. The follicular phase starts on the first day of the menstrual cycle and lasts until the end of ovulation (10–14 days). This phase is characterized by a gradual increase in the follicle-stimulating hormone, lower levels of progesterone, and a peak of estrogen close to ovulation. The luteal phase begins at the end of the ovulation until the next menstrual flow, with a rise in both estrogen and progesterone levels (Sherman and Korenman, 1975; Günther et al., 2015).

Estrogen concentration plays an important role across the menstrual cycle, modulating the brain activity, and emotional outcomes (e.g., mitigating negative mood responses) (Gonda et al., 2008; Albert et al., 2015). Moreover, the estrogen concentration was also observed to have a neuroexcitatory effect (Smith et al., 2002), that can be implicated in changes in athletic performance, however with no consensus based on previous studies (De Jonge et al., 2019; Meignié et al., 2021). The relationship between female athletes' performance and the menstrual cycle is a topic that has been extensively investigated and debated with contradictory findings (De Jonge et al., 2019; Carmichael et al., 2021a; Meignié et al., 2021). Moreover, to date, there is a gap in understanding the possible changes in athletes' subjective responses across the menstrual cycle. Addressing this important information on this topic can provide coaches with additional insight into how to better adapt training sessions while taking into account the influence of athletes' subjective response to training stimuli at different phases of the menstrual cycle. Therefore, the purpose of the present review and meta-analysis is to summarize the literature about the effect of different menstrual cycle phases on perceptual responses in female athletes. It is hypothesized that in phases with major ovarian hormones concentrations, an improvement of positive feelings and attenuation of negative ones will be perceived by the athletes.

## Methods

A systematic review with meta-analysis was performed under the guidelines of the Preferred Reporting Items for Systematic Reviews and Meta-Analyses (PRISMA) updated in 2020 (Page et al., 2021), and the protocol was registered at PROSPERO with the number CRD = 42022303166.

### Eligibility Criteria for Selecting Studies

Studies were eligible for inclusion following the PECO criteria: Participants (P): female athletes, from all sports disciplines, levels, and age categories. Exposure (E): at least two different menstrual phases assessment (e.g., follicular and luteal). Comparator (C): results of perceptual variables measured in two or more menstrual phases. Outcomes (O): maintenance or improvement of athletes' perceptual responses (e.g., exertion, fatigue, soreness, recovery, mood, wellbeing, motivation, anxiety, stress, and sleep) during one phase against another (online Supplementary Table 1). Studies were ineligible if the outcomes of interest were not measured or if the results were not reported in tables nor described in the manuscript. Non-English language articles, reviews or guidelines, letters to the editor, conference abstracts, and dissertation thesis were excluded. Moreover, data from women using oral contraception or menstrual dysfunction were not considered. Articles that reported data from women using oral contraception and women with normal cycle, data was extracted only from group of normal cycle. Articles published until January of 2022 were included.

### Search Strategy and Selection Process

A search strategy was performed on MEDLINE (*via* PubMed), SportDiscus (*via* EBSCO*host*), and Web of Science during the January of 2022. The search terms used PECO criteria and a full search of each database was performed according to MeSH descriptors with entry terms for the PubMed database. Following the descriptors with the Booleans operators: “menstrual cycle” AND “athlete” AND “perceptual responses” using the entry terms and derivative words related to perceptual measures (online Supplementary Table 1).

The articles from the databases were imported into the Rayyan systematic review software (Ouzzani et al., 2016) to proceed with the selection process. A multi-stage process was performed, as follows: (i) one reviewer (ACP) included in the Rayyan software the articles from each database; (ii) next, the same reviewer excluded the repeated, review letters to the editor and articles in non-English languages (identified by the software); (iii) two independent reviewers (MG, KD) screened the title and abstract and one reviewer checked the excluded articles in this phase (ACP); (iv) two independent reviewers (ACP, MG) screened the full text. Any disagreement between reviewers in phase iii were consulted by a third reviewer (ACP). As suggested previously (Garritty et al., 2021), a prior pilot selection with the 38 first articles was performed, demonstrating an agreement of 92% between the two reviewers (MG and KD with 3 disagreements).

### Data Collection Process and Assessment of Study Quality

An extraction form was developed for the reviewers' ACP and MG to extract data from each of the included studies. The extracted data included: the sample characteristics (e.g., size, sport modality and age), menstrual cycle phase (e.g., luteal, follicular, ovulation, and menstrual cycle measurement), perceptual measure (e.g., exertion, fatigue, soreness, recovery, mood, wellbeing, motivation, anxiety, stress, and sleep) and relevant outcomes (significant decrease, increase, or maintenance).

Study quality was assessed using the Downs and Black scale (1998), selecting relevant questions according to the selected study methodology (online Supplementary Table 3). The procedure was based on a previous review performed by Meignié et al. (2021) with a similar approach (e.g., menstrual cycle comparison in athletes). The original protocol consists of a checklist with items that include the study quality of reporting, internal and external validity and statistical power (Downs and Black, 1998). For each item, a binary score was used, in which 0 represents no/unable to determine and 1 = yes, an indication the study present the item. Due to the exclusion of some questions, the final score was converted to percentages and the methodological quality was classified as follows: <45.4% “poor” methodological quality; 45.4–61.0%, “fair” methodological quality; and >61.0%, “good” methodological quality (Meignié et al., 2021). The process was conducted by two independent reviewers (ACP and MG) and any disagreement was resolved by discussion.

### Statistical Analysis

The comprehensive Meta-Analysis software (CMA v2, Biostat, NY, USA) was used to calculate the difference in means (MD) in athletes' perceptual responses between different menstrual cycle phases. Statistical heterogeneity was assessed using *Q* and *I*^2^ statistics. The *I*^2^ was calculated to determine the degree of heterogeneity between included studies in each meta-analysis and was interpreted as follows: low (25%), moderate (50%), and high (75%) statistical heterogeneity (Higgins et al., 2003). Due to the high degree of heterogeneity between studies, a random-effects model was used for all comparisons. In addition, the publication bias was assessed by examining the asymmetry of the funnel plot using Egger's statistical test, considering a statistical bias to exist when *p* < 0.10. Moreover, a sensitivity analysis was performed by using both fixed and random effects models and by removing one study from the analysis. The significance level was set at *p* ≤ 0.05.

## Results

### Included Studies and Characteristics

**Figure 1:**
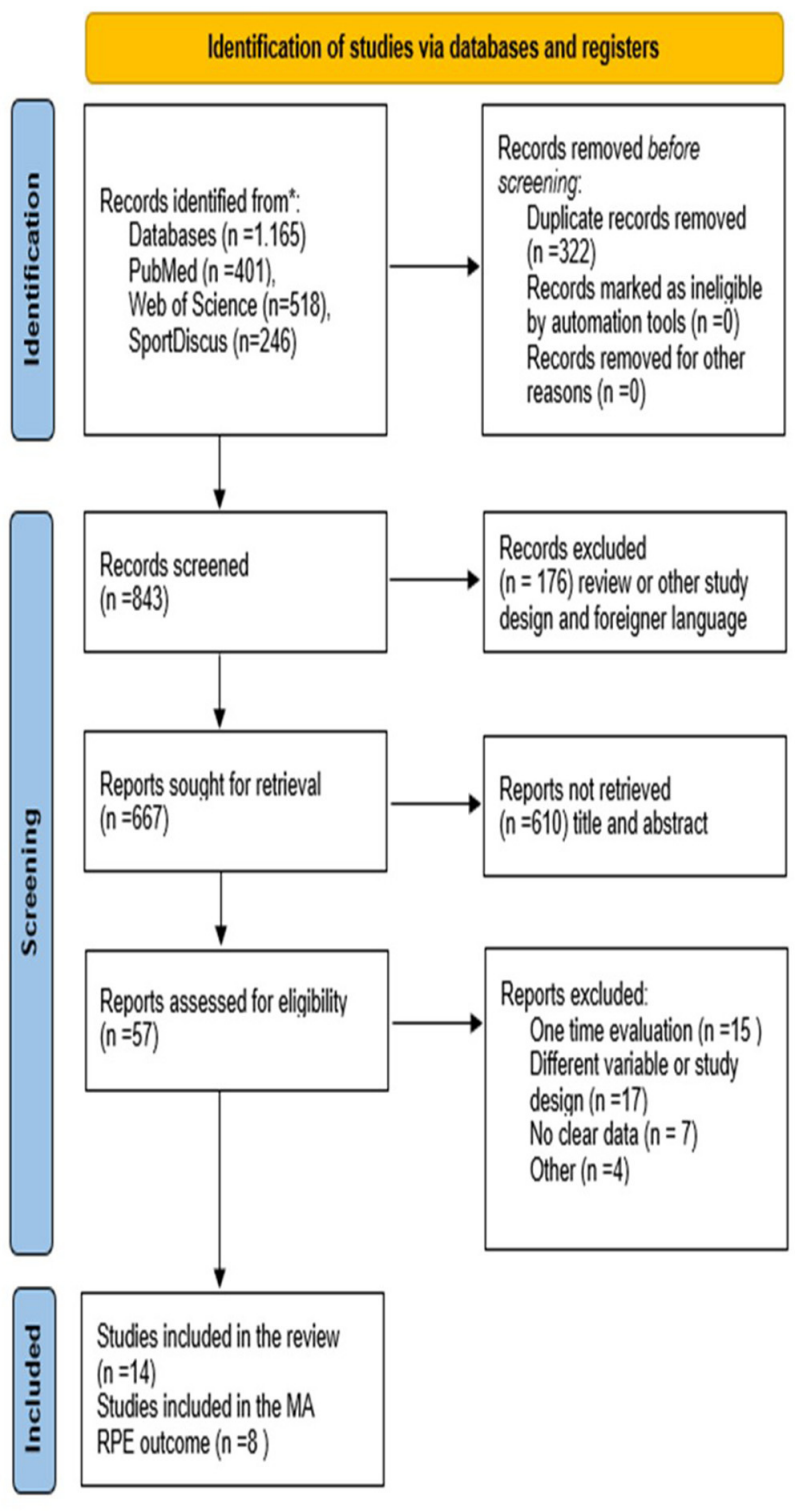
Flow chart diagram of the study process (PRISMA 2020).

A total of 1,165 records were found in searched databases. After duplicate removal, we screened 843 records, of which 176 studies were excluded for presenting a review method or foreign language. Therefore, 667 were retained to screen the title and abstract, of which 610 were excluded because they did not present a description of the menstrual cycle phase and perceptual measures in athletes. The last stage was to read the full text of the remaining 57 articles and after excluding the articles for the reasons of lacking information, 14 articles were included in the review. Later, we search documents that cited any of the initially included studies as well as the references; however, no extra articles that fulfilled inclusion criteria were found (Figure 1).

The methodological quality of the studies included, based on modified Downs and Black, showed that two studies were classified as “fair” methodological quality (Cockerill et al., 1992; Sunderland and Nevill, 2003) and the remaining articles were classified as having a “good” methodological quality ranging from 61.1% (Cook et al., 2018; Martínez-Cantó et al., 2018) to 83.3% (Chaffin et al., 2011; Julian et al., 2017; Graja et al., 2020; Carmichael et al., 2021b). The major limitation among the studies was the not representativeness of the participants from the entire population and the insufficient power to detect the menstrual cycle effect.

Table 1 summarizes the main characteristics of the included studies. The athletes included in the review are from endurance sports (runners, track and field, triathlete) and ball games (football, handball, rugby), varying from elite athletes to sport science students from different sports disciplines. Most studies monitored the athletes' menstrual cycle before the start of the data collection, ranging from 1 to 6 months previously. Early follicular (EF) and mid-luteal (ML) phases were the most phases investigated among the studies, based on the beginning of bleeding and after 18–22 days, respectively (online Supplementary Table 2). Four studies evaluate the perceptual responses during luteal, ovulation and follicular (Cook et al., 2018; Crewther and Cook et al., 2018; Cristina-Souza et al., 2019; Lara et al., 2020). The day of the menstrual cycle together with urine luteinizing hormone measurement were used as a method to establish the phases (De Souza et al., 1990; Graja et al., 2020; Lara et al., 2020; Carmichael et al., 2021b; Rael et al., 2021). One study evaluated two consecutive menstrual cycles (Cristina-Souza et al., 2019), two studies analyzed three consecutive menstrual cycle phases (Cook et al., 2018; Crewther and Cook, 2018), showing consistency in the outcomes across the cycles.

**Table 1:** Sample characteristics, menstrual cycle measurement and perceptual variable.

**Sample characteristic**			**Menstrual cycle measurement**				**Perceptual variable**	
**References**	**Sport modality sample size/age**	**Control group**	**Previous cycle monitoring**	**Phases comparison**	**Cycle length (days)**	**Measurement**	**Variable**	**Tool**
Carmichael et al. (2021b)	Elite football players *n* = 5/18–35 years	–	**–**	F/L	28 ± 3	Day control and LH urine test strip	Sleep, stress, soreness and fatigue; RPE	Wellness questionnaire; Borg CR-10
Chaffin et al. (2011)	Competitive runners *n* = 9/26.8 ± 4.3 years	**-**	3 months (Participant's documentation)	EF/ML	30.6 ± 4.6	Day control, estradiol and progesterone concentrations	Muscle soreness	DOMS rating scale
Cockerill et al. (1992)	Runners *n* = 13/29 years	Amenorrhoeic runners (*n* = 7); sedentary (*n* = 20)	**–**	PM/MC	–	Day control	Mood	POMS–TMD
Cook et al. (2018)	Elite athletes from different sports *n* = 6/21.0 ± 1.1 years	Non-elite athletes *n* = 16/21.1 ± 1.2 years	**–**	D7/D14/D21 (3 consecutive cycles)	24–33 (28.7 ± 1.9)	Day control	Motivation to train Motivation to compete	Likert scale 1–7 Likert scale 1–7
Crewther and Cook (2018)	Elite athletes from different sports *n* = 9/20.7 ± 1.3 years	Non-elite athletes *n* = 21/21.0 ± 1.1 years	**–**	F/O/L (3 consecutive cycles)	Cycle 1: 27.9 ± 0.8 Cycle 2: 28.1 ± 1.42 Cycle 3: 29.3 ± 1.8	Day control	Competitiveness Motivation to train	Likert scale 1–7 Likert scale 1–7
Cristina-Souza et al. (2019)	Track and field athletes *n* = 12/16.5 ± 1.6 years	**–**	2 months (Participant's documentation)	F/O/L	29 ± 3	Day control and hormonal analysis	RPE; MCS	Borg CR10; PMT-A, PMT-C, and PMT-D
De Souza et al. (1990)	Runners *n* = 8/29.0 ± 4.2 years	Amenorrhoeic runners *n* = 8/24.5 ± 5.7 years	1 month (LH urine test to determine the ovulatory period)	EF/ML	23–33	LH urine test strip	RPE	Borg 6–20 scale
Graja et al. (2020)	Handball players *n* = 10/20–25 years	–	6 months (menstruation diary)	F/L/PM	–	Day control, urine LH strip	Sleep, fatigue, stress, and soreness	Hooper index
Julian et al. (2017)	High-level soccer players *n* = 9/18.6 ± 3.8 years	–	6 months (menstruation diary)	EF/ML	–	Day control and serum hormones	RPE	Borg 6–20 scale
Lara et al. (2020)	Well-trained triathletes *n* = 13/31 ± 6 years	Placebo vs. caffeine	4 months (Mobile app Mycalendar)	EF/PO/ML	27 ± 2 days (24–31 days)	Day control, LH urine test strip	Self-perceived endurance; Exertion	1–10 scale; Borg 6–20 scale
Martínez-Cantó et al. (2018)	Physically active sport science students *n* = 5/20.33 ± 2.58 years	–	3 months (Participant's documentation)	M/L	31.20 ± 1.82	Day control	RPE Sleep Mood	Borg CR-10 KSD POMS
Miskec et al. (1997)	College club rugby players *n* = 10/21.9 ± 1.4 years	–	2 months (Participant's documentation)	M/Non-M	–	Day control	RPE	Borg 6–20 scale
Rael et al. (2021)	Well-trained in endurance activities *n* = 21/30.5 ± 6.5 years	**–**	6 months before the study—regular cycle	EF/LF/ML	28 ± 2–31 ± 2	Day control, urinary LH measurement and serum hormone analysis	RPE Perceived readiness	Borg 6–20; PR Nurmekivi 1–5 scale
Sunderland and Nevill (2003)	Well-trained game players *n* = 7/20.3 ± 0.3 years	OC well-trained game players *n* = 8/20.2 ± 0.4 years	**–**	F/L	24–30	Day control	RPE	Borg 6–20 scale

*EF, Early follicular; PO, Preovulation; ML, Mid luteal; LF, Late follicular; PM, Premenstrual; MC, Midclyce; F, Follicular; L, Luteal; MF, Mid follicular; M, Menstrual phase; Non-M, Nonmenstruation; LH, Luteinizing hormone; D7, day 7; D14, day 14; D2, day 21; OC, Oral contraception; RPE, Rating perceived effort; DOMS, Delayed onset muscle soreness; TRIMP, Training impulse; PMT-A(anxiety, nervous tension, irritability, mood changes); PMT-C (craving for sweets, headache, fatigue, fainting spells, palpitations); PMT-D (depression, withdrawal, insomnia, forgetfulness, and confusion); POMS, Profile of mood state; KSD, Karolinska sleep diary; TMD, Total mood disturbance*.

### Perceptual Variables and Outcomes

Perceptual outcomes were motivation (Cook et al., 2018; Crewther and Cook, 2018), competitiveness (Crewther and Cook, 2018), sleep quality (Martínez-Cantó et al., 2018; Graja et al., 2020; Carmichael et al., 2021b), stress (Graja et al., 2020; Carmichael et al., 2021b), muscle soreness (Chaffin et al., 2011; Graja et al., 2020; Carmichael et al., 2021b), fatigue (Graja et al., 2020; Carmichael et al., 2021b), perceived effort (De Souza et al., 1990; Miskec et al., 1997; Sunderland and Nevill, 2003; Julian et al., 2017; Martínez-Cantó et al., 2018; Cristina-Souza et al., 2019; Lara et al., 2020; Carmichael et al., 2021b; Rael et al., 2021), perceived endurance (Lara et al., 2020), mood (Cockerill et al., 1992; Martínez-Cantó et al., 2018), menstrual symptoms (Cristina-Souza et al., 2019), and readiness (Rael et al., 2021). Significant effect of menstrual cycle, described by the articles, was found on motivation, competitiveness, mood parameters, fatigue, sleep quality, and menstrual symptoms (Table 2).

**Table 2:** Perceptual measure and main outcomes.

**Study**	**Motivation**	**Competitiveness**	**Sleep**	**Stress**	**Soreness**	**Fatigue**	**RPE**	**Perceived Endurance**	**Mood**	**Menstrual symptoms**	**readiness**
Carmichael et al. (2021b)			↓L vs. F	↔	↔	↑ L vs. F	↔				
Chaffin et al. (2011)					↔						
^a^Cockerill et al. (1992)									↑PM vs. MC (88.1%)		
^b^Cook et al. (2018)	↑ D14 vs. D7, D2										
Crewther and Cook (2018)	↑ O vs. F, L	↑ O vs. F, L ↑ F vs. L									
Cristina-Souza et al. (2019)							↔			↑F vs. O	
De Souza et al. (1990)							↔				
Graja et al. (2020)			↔	↔	↔	↔					
Julian et al. (2017)							↔				
Lara et al. (2020)							↔	↔			
Martínez-Cantó et al. (2018)			↔				↔		↓M vs. L (vigor) 22.7%		
Miskec et al. (1997)							↔				
^c^Rael et al. (2021)							↔				↑ EF vs. LF and ML (6.1%)
Sunderland and Nevill (2003)							↔				

*↑, significant increase/improvement; ↓, significant decrease/impairment; ↔, no significant difference*.

*^a^Significant low TMD score of the non-amenorrhoeic runners in PM and MC compared to amenorrhoeic runners and sedentary.*

*^b^The same result from motivation to train and motivation to compete.*

*^c^P=0.011 but post-hoc reported no significant differences*.

Motivation was assessed in two studies (Cook et al., 2018; Crewther and Cook, 2018) and competitiveness in one study (Crewther and Cook, 2018) in athletes from different sports, presenting higher values during the ovulation phase compared to follicular and luteal. Parameters of mood also were evaluated in two studies: one study with runners presenting a significant high mood disturbance during the premenstrual phase (later luteal) compared to middle-cycle (mid-luteal) (Cockerill et al., 1992) and one study with sport science students demonstrating a significant decrease on vigor during the menstrual period (early follicular) compared to luteal phase (day 18–20 days) (Martínez-Cantó et al., 2018). Menstrual symptoms, reported by one study, were significantly higher during the follicular compared to the ovulation phase in track and field athletes (Cristina-Souza et al., 2019).

Sleep quality was assessed in three studies. Only one demonstrated a significant decrease of quality during the luteal phase compared to the follicular phase, performed in elite football players (Carmichael et al., 2021b). Fatigue was assessed in two studies, with one study, presenting a significant perception of fatigue during the luteal vs. follicular phase (Carmichael et al., 2021b). Perceived effort was evaluated in 9 studies, and all of them reported no significant difference between the menstrual cycle phases (De Souza et al., 1990; Miskec et al., 1997; Sunderland and Nevill, 2003; Julian et al., 2017; Martínez-Cantó et al., 2018; Cristina-Souza et al., 2019; Lara et al., 2020; Carmichael et al., 2021b; Rael et al., 2021).

### Results From the Meta-Analysis

In order to perform the meta-analysis, it was considered studies that presented outcomes of perceptual variables measured by the same tool. The perceived of exertion was evaluated in most of the studies, using the Borg Scale (e.g., 6–20 and 0–10 points). Therefore, 0–10 points scale was normalized to 6–20 points scale. It was opted to analyze the effect of menstrual phase based on the day of the cycle instead of the name of the phases provided by the studies. Based on days evaluated in the studies, and the well-defined changes in ovarian hormones concentration on those days (online Supplementary Table 2), the meta-analysis was performed for comparison of day 1–5 vs. day 19–24 only.

**Figure 2:**
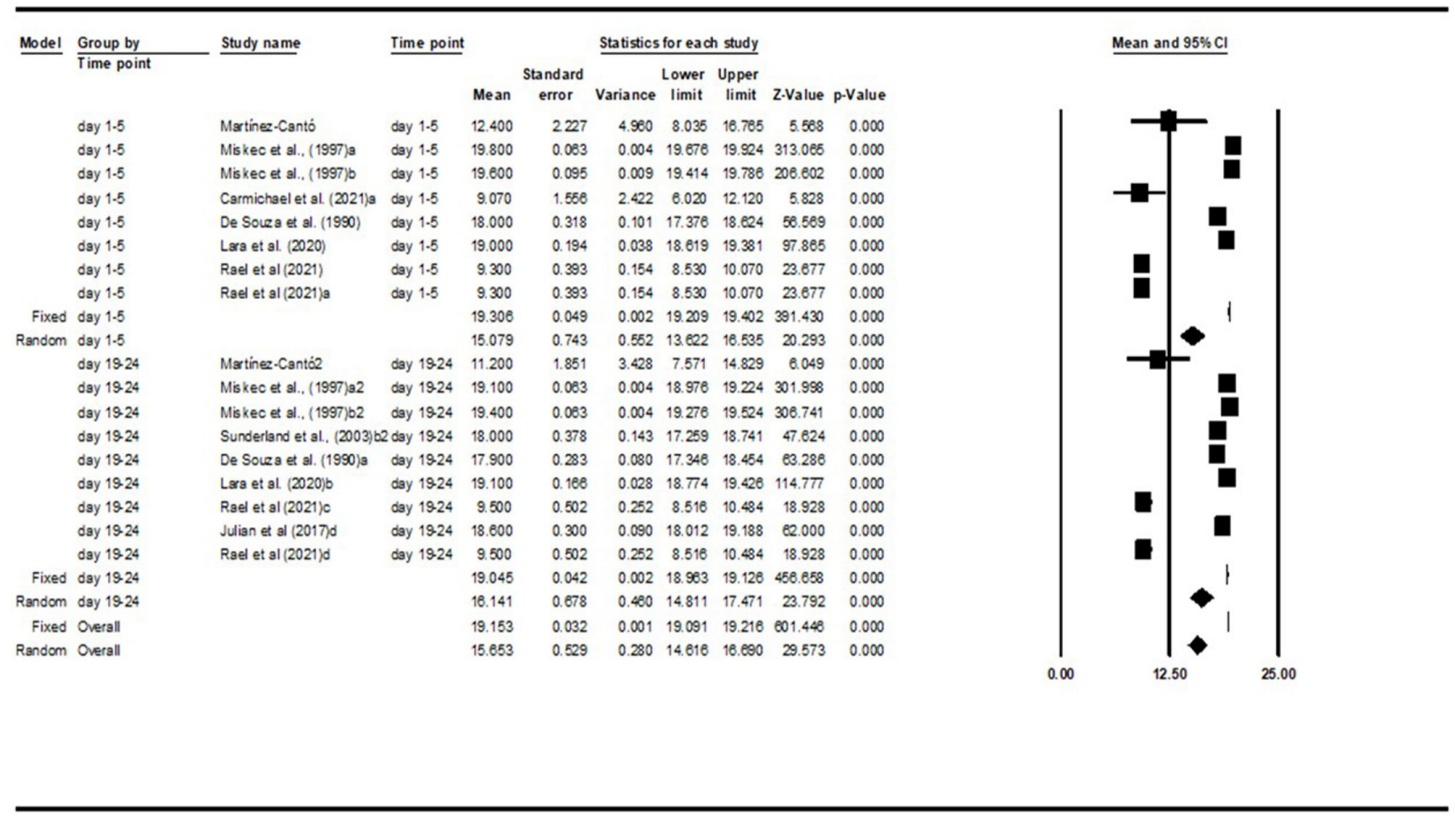
Meta-analysis.

Our results showed that level of perceived exertion does not differ between two phases of the menstrual cycle (*MD* = −1.36, *Q* = 1.11, *df* = 1, *p* = 0.209), whereas RPE was 15.08 ± 0.74 and 16.14 ± 0.68 at day 1–5 and day 19–24, respectively. When one study was removed, a similar results were observed (*MD* = −0.48; *Q* = 0.22; *p* = 0.639) (Figure 2).

## Discussion

The present review aimed to investigate the effect of perceptual responses during the menstrual cycle, with a hypothesis that in phases with major ovarian hormones concentration, an improvement of positive feelings and attenuation of negative ones will be perceived by athletes. As the main findings, the current review identified that some perceptual responses were better during ovulation (e.g., motivation and competitiveness) compared to luteal or follicular phase. Negative perceptual responses were exacerbated during pre-menstruation (e.g., mood disturbance) and during menstruation period (e.g., menstrual symptoms and drop in vigor) compared to luteal and ovulation phases. Other responses such as perceived effort, stress and muscle soreness were not found to be affected by the menstrual cycle.

The effect of the menstrual cycle on athletes' responses still a sensitive area to interpret, as there are only a limited number of original studies with a heterogeneous methodology, differing in the type of variables used and methodology applied to determine menstrual phases. In the present review, the ovulation period showed to play a key role in the positive response to motivation and desire to compete, as demonstrated in the Cook and Crewther studies (Cook et al., 2018; Crewther and Cook, 2018). Moreover, the authors also described changes in salivary testosterone concentrations across the menstrual cycle [higher concentration in ovulation (day 14) compared to follicular (day 7) and luteal (day 21) phases] and highlighted that these changes were positively accompanied by an increase in the motivation to train and to compete. Indeed, it was previously demonstrated that testosterone concentrations rise from the follicular to the ovulatory phase and then fall during the luteal phase (Cook et al., 2021). The increase in testosterone concentration supports positive athletic behavior such as self-efficacy, pre-event focus, motivation for action, team bonding, and self-chosen workloads in athletic and non-athletic domains, aggressiveness, and risk-taking (Cook et al., 2018; Crewther and Cook, 2018).

A positive relation between ovulation and perceptual responses has been demonstrated earlier, in which women seem to present an increase in libido, energy, and competitiveness with mates (Motta-Mena and Puts, 2017). Therefore, it is reasonable to consider the period close to ovulation as an advantage for female athletes, to maximize their performance in training settings. From a practical point of view, the coaches and practitioners can seize the opportunity to adjust the athletes' training load and/or intensity which could lead to a major training adaptation. All the same, Julian and Sargent (2020) suggested some practical and future research considerations of a training periodization based on the menstrual cycle phase. The authors describe the importance of investigating the relationship between menstrual hormones and training responses in a longitudinally designed manner, in order to verify a possible effect. However, it could be an expensive and impractical venture for small clubs. Validation of practical and cost-effective methods for monitoring hormone levels (e.g., calendar-calculation vs. hormone concentration), particularly for ovulation detection, can help optimize training periodization and link athletes' perceptual responses to training performance.

As described earlier, estrogen can act as a protective factor against negative mood responses (Albert et al., 2015), thus, in phases with a low concentration, negative feelings can be potentiated. In the current review, athletes reported an increase in mood disturbance during the pre-menstrual period compared to the middle of the cycle (Cockerill et al., 1992). Also, a reduction in vigor (Martínez-Cantó et al., 2018) and an increase of menstrual symptoms (Cristina-Souza et al., 2019) were found during the menstrual period compared to the mid-luteal and ovulatory phases, respectively. This support previous findings that female athletes perceived the period of menses with a feeling of discomfort, pain and disturbance in mood (Brown et al., 2021). The loss of vigor found in the Martínez-Cantó et al. (2018) study could be related to negative feelings such as the menstrual symptoms found in Cristina-Souza et al. (2019) study. The negative perceptual outcomes might impact the athletes' performance, affecting negatively their capacity to train, by an increase in training monotony and strain (Cristina-Souza et al., 2019). Similarly, a narrative review described that athlete perceived impairment on their performance during the early follicular and late luteal phases compared to the rest of the menstrual cycle (Carmichael et al., 2021b).

Although the link between negative symptoms and early follicular and later luteal phase founded in some studies, others reported no difference in subjective outcomes such as sleep, stress, muscle soreness, fatigue, and perceived effort. Only the study of Charmichael et al. (2021), which evaluated five elite athletes from one football team, described a decline in subjective sleep quality and increase in fatigue in the luteal phase compared to the follicular phase. The authors speculate that the greater fatigue perceived in the luteal phase could be due to the lower sleep quality also reported in this phase. Interestingly, the athletes presented negative outcomes not in the menses period (with lower ovarian concentration), but 14 days before the onset of menstrual bleeding (approximately after the ovulation period). As described by the authors, the small sample size and the menstrual phase determination could bias the results observed.

Lastly, regardinds the perceived efforts, when the data was organized by days of the cycle, an RPE of 19.81 ± 0.05 was observed on days 1–5 and an RPE of 16.27 ± 0.53 was observed on days 19–24. Althought non-significant difference in RPE was found, athletes presented a major effort at the beginning of the cycle compared to middle luteal phase. Subsequent studies found an increase in RPE given exercise intensity during the follicular phase (menses period), which may reduce the capacity to perform the exhaustive exercise (Janse de Jonge et al., 2012; Mattu et al., 2020). Speculative explanation for the findings can be related to high methodological heterogeneity between included studies in the meta-analysis. The two-phases comparison, and the exclusion of ovulation phase masked possible changes across the menstrual cycle. A long-term monitoring of RPE during the training program could better reflect the effect of menstrual cycle on athletes' perception of training session intensity.

### Limitations of the Study

Although the present review was the first to examine the effects of the menstrual cycle on perceptual responses in athletes, there are some limitations that must be emphasized. The diverse perceptual variables measured and different phases of menstrual cycle evaluated amongst the studies could be the major limitation to the generalization of the results. The use of RPE measure in different test protocol limitate the interpretation of the efforce. The lack of criteria to define the phases is a problem addressed previously, making the studies involving menstrual cycle effect difficult to compare or establish some inference. The small sample size in most of articles also increase the caution in generalizing the results, and the outcomes from perceptual measures of only one study should be taken into consideration.

## Conclusion

The hormonal fluctuation across the menstrual cycle and the link with athletes' subjective responses are still a topic ambitious to interpret, as the studies present insufficient power to identify the effects. Similar to the previous review about menstrual cycle and performance, the variety of perceptual responses evaluated and the different methodological approach, limiting the generalization of the conclusion. Nonetheless, the current review identifies that some perceptual responses were better during ovulation (e.g., motivation and competitiveness) than the late luteal or mid follicular phases. Negative perceptual responses were exacerbated pre-menstruation (e.g., mood disturbance) and during the menstruation period (e.g., menstrual symptom and drop on vigor) than mid-luteal and ovulation phases. Other responses were not observed to be influenced by the menstrual cycle (e.g., perceived effort, stress and muscle soreness).

From the studies that did observe a statistically significant effect of the menstrual cycle phase, it can be noticed that a “favorable” subjective response may be find in the late follicular to ovulatory phase in athletes. However, the outcomes that come from the one phase should be taken into consideration, due to the variability of the phases assessed in the studies. Establishing a battery of subjective and objective measurement and monitoring the daily responses together with training or competition demands across the menstrual cycle might provide effective feedback to coaches and athletes, in order to develop strategies inside the training programming training to manage both the athlete's performance, health and wellbeing.

## Data Availability Statement

The original contributions presented in the study are included in the article/Supplementary Material, further inquiries can be directed to the corresponding author/s.

## Author Contributions

ACP and MG: conceptualization and search. ACP, KD, and MG: data selection. AP: data analysis. ACP, MG, and AP: drafted manuscript. All authors critically revised the manuscript, contributed to the article, and approved the submitted version.

## Funding

This project was supported by the Specific University Research Grant provided by the Ministry of Education, Youth, and Sports of the Czech Republic (number MUNI/A/1389/2021). The author ACP was support by the Evaluation of Graduate Education, and from Operational Programme Research, Development, and Education-Project Postdoc2MUNI (Grant No. CZ.02.2.69/0.0/0.0/18_053/0016952).

## Conflict of Interest

The authors declare that the research was conducted in the absence of any commercial or financial relationships that could be construed as a potential conflict of interest.

## Publisher's Note

All claims expressed in this article are solely those of the authors and do not necessarily represent those of their affiliated organizations, or those of the publisher, the editors and the reviewers. Any product that may be evaluated in this article, or claim that may be made by its manufacturer, is not guaranteed or endorsed by the publisher.
